# Zebrafish is a predictive model for identifying compounds that protect against brain toxicity in severe acute organophosphorus intoxication

**DOI:** 10.1007/s00204-016-1851-3

**Published:** 2016-09-21

**Authors:** Melissa Faria, Eva Prats, Francesc Padrós, Amadeu M. V. M. Soares, Demetrio Raldúa

**Affiliations:** 1 Centre of Environmental and Marine Studies (CESAM), University of Aveiro, 3810-193 Aveiro, Portugal; 2 CID-CSIC, Jordi Girona 18, 08034 Barcelona, Spain; 3 IDAEA-CSIC, Jordi Girona 18, 08034 Barcelona, Spain; 4 Fish Diseases Diagnostic Service, Facultat de Veterinaria, Universitat Autònoma de Barcelona, 08190 Bellaterra (Cerdanyola del Vallès), Spain

**Keywords:** Zebrafish model, Severe acute organophosphorus intoxication, Brain toxicity, Neuroprotection, Antidotes

## Abstract

Acute organophosphorus (OP) intoxication is a worldwide clinical and public health problem. In addition to cholinergic crisis, neurodegeneration and brain damage are hallmarks of the severe form of this toxidrome. Recently, we generated a chemical model of severe acute OP intoxication in zebrafish that is characterized by altered head morphology and brain degeneration. The pathophysiological pathways resulting in brain toxicity in this model are similar to those described in humans. The aim of this study was to assess the predictive power of this zebrafish model by testing the effect of a panel of drugs that provide protection in mammalian models. The selected drugs included “standard therapy” drugs (atropine and pralidoxime), reversible acetylcholinesterase inhibitors (huperzine A, galantamine, physostigmine and pyridostigmine), *N*-methyl-d-aspartate **(**NMDA) receptor antagonists (MK-801 and memantine), dual-function NMDA receptor and acetylcholine receptor antagonists (caramiphen and benactyzine) and anti-inflammatory drugs (dexamethasone and ibuprofen). The effects of these drugs on zebrafish survival and the prevalence of abnormal head morphology in the larvae exposed to 4 µM chlorpyrifos oxon [1 × median lethal concentration (LC_50_)] were determined. Moreover, the neuroprotective effects of pralidoxime, memantine, caramiphen and dexamethasone at the gross morphological level were confirmed by histopathological and transcriptional analyses. Our results demonstrated that the zebrafish model for severe acute OP intoxication has a high predictive value and can be used to identify new compounds that provide neuroprotection against severe acute OP intoxication.

**Electronic supplementary material:**

The online version of this article (doi:10.1007/s00204-016-1851-3) contains supplementary material, which is available to authorized users.

## Introduction

Organophosphorus (OP) compounds are a class of acetylcholinesterase (AChE) inhibitors used not only in agriculture and industry but also as chemical warfare nerve agents. Severe acute OP intoxication is a worldwide clinical and public health problem, with an estimated 3 million cases and 300,000 deaths annually (Bertolote et al. 2006; Eddleston and Phillips 2004). In developing countries, in particular those from the Asia–Pacific region, the major concern is self-poisoning with OP pesticides. However, developed countries are predominantly concerned with the potential use of highly toxic OP compounds by terrorists or the release of these compounds during transportation or from storage facilities after an accident or natural disaster (Jett and Yeung 2015).

Neurodegeneration and brain damage are the hallmarks of severe acute OP intoxication. OP compounds inhibit AChE, resulting in the accumulation of the neurotransmitter acetylcholine (ACh) at the cholinergic synaptic clefts and subsequent long-term activation of the nicotinic and muscarinic ACh receptors (AChR), overstimulation of the cholinergic neurons, hyperexcitation and seizures (Pena-Llopis 2005). Then, a cascade of downstream events occurs, resulting in secondary neuronal toxicity. The release of excitatory amino acids (EAAs), such as glutamate and aspartate, and the activation of the *N*-methyl-d-aspartate (NMDA) receptors promote intracellular Ca^2+^ influx, which can activate different lipases, proteases, endonucleases, kinases or phosphatases and result in severe brain damage (Kaur et al. 2014). The generation of reactive oxygen or nitrogen species may also play an important role in the development of neuroinflammation and cellular death that are found in severe acute OP intoxication (Eisenkraft et al. 2013; Pena-Llopis 2005).

Although many different mechanisms are involved in the pathophysiology of severe acute OP intoxication, the standard therapy has not changed much over the last 50 years. Pyridostigmine bromide is the only FDA-approved prophylactic drug (Jett and Yeung 2015), and standard therapy is essentially restricted to the administration of atropine to counteract muscarinic overstimulation and an oxime to reactivate AChE (Balali-Mood and Saber 2012). Administration of benzodiazepines to control convulsions and mechanical respiration may be required. However, the limitations of these treatments are well known, and new and more efficient therapies are needed (Albuquerque et al. 2006; Buckley et al. 2004).

Zebrafish is a vertebrate model increasingly used in biomedical research, including human toxicology studies (Raldúa et al. 2012; Thienpont et al. 2011). One key advantage of zebrafish embryos/larvae over other vertebrate models for drug discovery is their suitability for in vivo high-throughput screening of chemical libraries for pharmacological and/or toxicological effects. In this context, zebrafish has been proposed as an intermediate step between single cell-based assays and mammalian (and ultimately human) testing. Furthermore, previous studies have indicated that zebrafish is an excellent organism for modelling human neuropathological processes (Babin et al. 2014; Kabashi et al. 2010).

Recently, we generated a zebrafish chemical model of severe acute OP intoxication using chlorpyrifos oxon (CPO) as a prototypic OP compound (Faria et al. 2015). At the gross morphological level, this zebrafish model was characterized by a compacted head with areas of opacification, which indicates brain necrosis (Rodriguez and Driever 1997). Further histopathological analyses confirmed the presence of severe brain damage underlying the observed morphological changes (Faria et al. 2015). Moreover, we demonstrated that the zebrafish severe acute OP intoxication model displays many of the pathophysiological mechanisms, including AChE inhibition, NMDA receptor activation, calcium dysregulation and activation of inflammatory and immune responses, underlying this toxidrome in humans. Three hours after exposure to CPO, a percentage of the larvae displayed morphological changes in the head, and the development of this larval phenotype was already irreversible. Although the above data strongly suggest that this model could be useful for identifying new compounds that protect against brain toxicity in humans with severe acute OP intoxication, additional studies are needed to demonstrate the predictive power of the model.

The purpose of this study was to assess the suitability of the zebrafish severe acute OP intoxication model for identifying new compounds that provide neuroprotection against severe acute OP intoxication in humans. We used this zebrafish model, which was induced with 4 μM CPO [1 × LC_50_ (median lethal concentration)], to assess the potential neuroprotective effects of a panel of drugs commonly used in human medicine (Table S1). First, *a pre*-*treatment therapeutic approach* was designed to assess the suitability of the zebrafish model for identifying medical countermeasures that protect against intoxication when administered prior to acute OP exposure. Personnel that should be pre-treated with these medical countermeasures include first responders, such as emergency medical technicians, and individuals responsible for site decontamination (Jett and Yeung 2015). Four reversible AChE inhibitors (huperzine A, galantamine, physostigmine and pyridostigmine), as well as the muscarinic AChR antagonist atropine and the oxime pralidoxime, were tested using the pre-treatment approach. Moreover, a *post*-*treatment therapeutic approach* was designed to assess the suitability of the model for identifying new molecules with neuroprotective effects in cases of severe acute OP intoxication. Atropine, pralidoxime and a panel of drugs targeting selected key events of the pathophysiological pathways of this condition were tested using the post-treatment approach. These selected drugs included two NMDA receptor antagonists (MK-801 and memantine), two dual-function NMDA receptor and AChR antagonists (caramiphen and benactyzine) and two anti-inflammatory drugs (dexamethasone and ibuprofen). The effects on the 24-h survival and the prevalence of abnormal heads were determined for all compounds. Moreover, the effectiveness of the countermeasures to protect the brain was further confirmed by histopathological evaluation and by mRNA quantification of three selected genes (*il*-*12*, *hspb11*, *pth1a*) that are potentially involved in severe acute OP intoxication. Our results demonstrate that the zebrafish model for severe acute OP intoxication provides reasonably accurate evaluations of the neuroprotective effects of well-characterized antidotes in mammalian models.

## Methods

### Fish husbandry and larvae production

Adult wild-type zebrafish were maintained in fish water [reverse-osmosis purified water containing 90 µg/ml of Instant Ocean (Aquarium Systems, Sarrebourg, France) and 0.58 mM CaSO_4˙_2H_2_O] at 28 ± 1 °C in the Research and Development Centre of the Spanish Research Council (CID-CSIC) facilities under standard conditions. Embryos were obtained by natural mating and maintained in fish water at 28.5 °C. Larvae were not fed during the experimental period. All procedures were conducted in accordance with the institutional guidelines under a licence from the local government (DAMM 7669, 7964) and were approved by the Institutional Animal Care and Use Committees at the CID-CSIC.

### Chemicals

Chlorpyrifos oxon (CPO) (CAS#5598-15-2, 98.1 % purity) was purchased from Chem Service (West Chester, PA). Galantamine hydrobromide (CAS#1953-04-4, ≥98 % purity) and benactyzine hydrochloride (CAS#57-37-4, ≥98 % purity) were purchased from Santa Cruz Biotechnology (Santa Cruz, CA). Atropine (CAS 51-55-8, ≥99 % purity), pralidoxime chloride (2-PAM; CAS#51-15-0, ≥97 % purity), (±)-huperzine A (CAS#120786-18-7, ≥ 98 % purity), caramiphen hydrochloride (CAS#125-85-9, ≥98 % purity), dexamethasone (CAS#50-02-2, ≥98 % purity), physostigmine (eserine hemisulfate salt; CAS#64-47-1, ≥99 % purity), memantine hydrochloride (CAS#41100-52-1, ≥98 % purity), pyridostigmine bromine (CAS#101-26-8, ≥98 % purity), MK-801 (CAS#77086-22-7, ≥98 % purity) and ibuprofen (CAS#15687-27-1, ≥98 % purity) were all purchased from Sigma-Aldrich (St. Louis, MO).

Stock solutions of CPO and dexamethasone were prepared in dimethyl sulfoxide (DMSO), stock solutions of ibuprofen were prepared in ethanol, and stock solutions of galantamine, benactyzine, atropine, pralidoxime, huperzine A, caramiphen, physostigmine, memantine, pyridostigmine and MK-801 were prepared directly in fish water. Exposure solutions were prepared by diluting the stock solution in fish water. The final concentration of the solvent in the exposure solutions was 0.01 %, except for the reversible AChE inhibitors. Several of these compounds only weakly penetrate the skin of zebrafish larvae in water (Behra et al. 2004; Fischer et al. 2015); thus, 1 % DMSO was added to the exposure medium for this group of compounds (Berghmans et al. 2008). A preliminary range-finding test was performed for each drug, and the final selected concentration corresponds to the no observed effect concentration (NOEC) for survival and gross morphological defects, unless otherwise stated.

### General experimental design

#### Severe acute OP intoxication model generation

For the severe acute OP intoxication model generation, zebrafish larvae were transferred to 48-well plates (1 larva per well) at 7 days post-fertilization (dpf) and exposed for 24 h to 4 µM CPO, which corresponds to 1 × LC_50_ (Faria et al. 2015), in a dark incubator at 28.5 °C. Control larvae were exposed to the same concentration of the carrier (0.1 % DMSO) under identical conditions. The zebrafish model was characterized by a compacted head with areas of opacification at the gross morphological level. At the end of every experiment, survival and prevalence of the morphological changes in the head were determined.

#### Therapeutic approaches

**Fig. 1:**
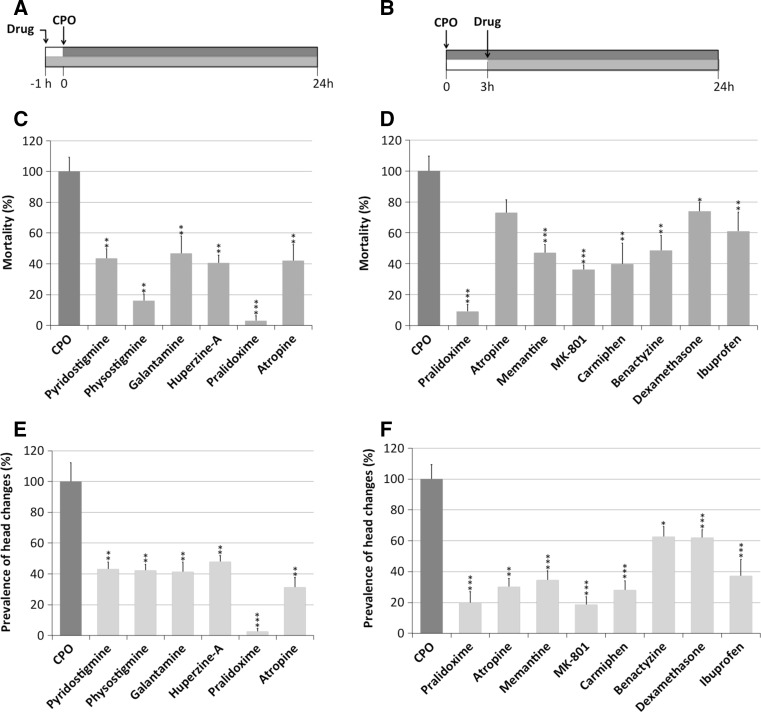
Drugs used in mammalian models to protect against severe acute OP intoxication have a similar effect in zebrafish. **a**, **b** Scheme of the pre-treatment (**a**) and post-treatment (**b**) experimental approaches used in this study to assess the effects of drugs administered for prophylaxis and treatment, respectively. **c**, **d** Effects of a panel of drugs on the mortality rate of the zebrafish severe acute OP intoxication model using the pre-treatment (**c**) and post-treatment (**d**) approaches. Mortality (%) for each drug is represented as the percentage of dead larvae (mean ± SE; *n*: 95–192) relative to that of the group exposed to 4 μM chlorpyrifos oxon (CPO) alone. Drug concentrations used in the pre-treatment approach were as follows: pyridostigmine, 10 mM; physostigmine, 75 μM; galantamine, 0.5 mM; huperzine A, 1 μM; pralidoxime, 0.4 mM; atropine, 0.4 mM. **e**, **f** Effects of a panel of drugs on the prevalence of the changes in the head morphology of the larvae using the pre-treatment (**e**) and post-treatment (**f**) approaches. Prevalence of the morphological changes (%) is represented as the percentage of larvae (mean ± SE; *n*: 80–192) exhibiting altered head morphology relative to that of the larvae exposed to CPO alone. Drug concentrations used in the post-treatment approach were as follows: pralidoxime, 0.4 mM; atropine, 0.4 mM; memantine, 100 μM; MK-801, 50 μM; caramiphen, 25 μM; benactyzine, 50 μM; dexamethasone, 40 nM; ibuprofen, 2.5 μM. The results are pooled data from 2 to 3 independent experiments. *Asterisks* indicate significant differences between the larvae treated with a drug and those in the CPO group [*P < 0.05; **P < 0.01 or ***P < 0.001, following a one-tailed Student’s *t* test]

Two different therapeutic approaches were used (see Fig. 1a, b):

1. *Pre*-*treatment* 7-dpf zebrafish larvae were pre-treated with selected concentrations of different prophylactic drugs for 1 h and were then co-exposed to a cocktail of 1 × LC_50_ CPO plus the prophylactic drugs for an additional 24 h. The drugs assessed using this therapeutic approach were galantamine (0.5 mM), huperzine A (1 µM), physostigmine (75 µM), pyridostigmine (10 mM), atropine (0.4 mM) and pralidoxime (0.4 mM). For galantamine, which has a very low permeability in zebrafish, the pre-treatment period was increased to 24 h (from 6 to 7 dpf). Survival and morphological features were recorded at the end of the 24 h incubation period.
2. *Post*-*treatment* 7-dpf zebrafish larvae were first challenged with 1 × LC_50_ CPO alone for 3 h and were then co-exposed for an additional 21 h to a cocktail of 1 × LC_50_ CPO plus the post-treatment drugs. The selected drugs included pralidoxime (0.4 mM), atropine (0.4 mM), MK-801 (100 µM), memantine (50 µM), caramiphen (25 µM), benactyzine (50 µM), dexamethasone (40 nM) and ibuprofen (2.5 µM). Survival and prevalence of the morphological changes in the head were assessed at the end of the incubation period (3 h + 21 h).

### Gross morphological analyses

Morphological analyses of the zebrafish head were performed using standard protocols (Supplementary Methods).

### Histopathological analysis

Histopathological analysis was performed using light microscopy with standard protocols (Supplementary Methods).

### RNA preparation and qRT-PCR analysis

RNA preparation and qRT-PCR analysis were performed following standard protocols (Supplementary Methods).

### Data analysis

Each experiment was carried out with its corresponding negative control (solvent control) and positive control (medium with CPO only). The two endpoints, survival and changes in head morphology, were calculated as a per cent of the total *n* of the corresponding treatment. The mean value of the CPO only responses was considered 100 %, and the results of all co-exposure treatments were then determined relative to the corresponding CPO treatment. The data were analysed with a Student’s *t* test using IBM SPSS 19.0 (Statistical Package 2010, Chicago, IL). Data are presented as the mean ± SEM of 2–3 independent experiments, unless otherwise stated. Significance was set at *P* < 0.05. Analysis of the qRT-PCR data, which was normally distributed (Levene’s test), was performed using the ΔΔ*Ct* method. Differences among the control and treated groups were analysed by Student’s *t* test.

## Results

### Pre-treatment

Using the pre-treatment therapeutic approach (Fig. 1a), we explored the potential prophylactic effects of four reversible AChE inhibitors (galantamine, huperzine A, physostigmine and pyridostigmine) on zebrafish larvae exposed to 1 × LC_50_ CPO for 24 h. Pre-treatment with all four drugs resulted in a significant reduction (*P* < 0.01) in both mortality and prevalence of larvae with morphological changes in the head (Fig. 1c, e; Table 1). The reduction in morphological changes was very similar across all tested drugs and had a range of 51.9–58.5 % (Fig. 1c, e; Table 1). However, there were differences among the drugs in the reduction of lethality. Thus, physostigmine increased the survival to 83.9 %, while the increases in survival caused by pyridostigmine, huperzine A and galantamine were more moderate (Fig. 1c; Table 1).

**Table 1:** Analysis of the effectiveness of twelve human antidotes to protect zebrafish larvae from severe acute OP intoxication

	Drug class	Exposure type	Total *n* ^a^	Phenotype prevalence %	*P* value	Mortality %	*P* value
Pralidoxime (0.4 mM)	Standard antidotes	Pre-treatment	192 (4)	2.65 ± 1.80	1.19E-05**	3.10 ± 3.09	3.72E-07**
Pralidoxime (0.4 mM)		Post-treatment	144 (3)	19.74 ± 7.2	1.61E-04**	9.28 ± 4.51	3.25E-06**
Atropine (0.4 mM)		Pre-treatment	95 (2)	31.4 ± 15.0	5.00E-04**	16.8 ± 10.4	1.80E-03**
Atropine (0.4 mM)		Post-treatment	95 (2)	30.3 ± 5.10	1.36E-03**	73.1 ± 8.25	6.04E-02
Pyridostigmine (10 mM)	AChE reversible inhibitors	Pre-treatment	131 (3)	43.1 ± 4.6	1.69E-05**	43.6 ± 6.13	3.24E-04**
Huperzine A (1 µM)		Pre-treatment	96 (2)	48.1 ± 3.8	6.17E-05**	40.6 ± 5.14	1.44E-04**
Galantamine (0.5 mM)		Pre-treatment	120 (2)	41.5 ± 16.4	3.12E-04**	46.8 ± 29.7	3.64E-03**
Physostigmine (75 µM)		Pre-treatment	126 (3)	42.4 ± 4.0	1.74E-04**	16.1 ± 4.25	6.21E-06**
Memantine (100 µM)	NMDA receptor antagonists	Post-treatment	191 (3)	34.6 ± 19.3	1.14E-07**	47.0 ± 16.7	7.93E-05**
MK-801 (50 µM)		Post-treatment	96 (2)	18.6 ± 11.13	2.31E-07**	36.1 ± 6.53	1.80E-04**
Benactyzine (50 µM)	AChR and NMDA receptor antagonists	Post-treatment	80 (2)	62.8 ± 6.50	1.08E-02*	48.7 ± 9.73	4.41E-03**
Caramiphen (25 µM)		Post-treatment	144 (2)	28.0 ± 19.4	1.32E-06**	39.9 ± 13.58	9.36E-03**
Ibuprofen (2.5 µM)	Anti-inflammatory	Post-treatment	95 (2)	37.2 ± 10.6	6.34E-06**	61.1 ± 12.27	7.31E-03**
Dexamethasone (40 nM)		Post-treatment	95 (2)	62.1 ± 4.98	3.68E-04**	73.9 ± 5.54	3.27E-02*

The prevalence of morphological changes in the head and mortality are represented as % relative to the corresponding CPO group. The results are shown as the mean ± SEM. *P* values are given for each endpoint and treatment, with * *P* < 0.05; ** *P* < 0.01, simple one-tailed Student’s *t* test

* *P* < 0.05; ** *P* < 0.01

^a^Number of experimental replicates

Atropine and/or pralidoxime are antidotes commonly administered in mammalian models of acute OP intoxication shortly before or 1 min after the OP exposure to reduce mortality. When zebrafish larvae were pre-treated with atropine, a significant increase in survival was found, with a concomitant reduction in the prevalence of morphological changes (Fig. 1c, e; Table 1). Interestingly, pre-treatment with pralidoxime provided the highest degree of protection of all the chemicals and treatments tested in this study, with a 97.4 ± 1.8 % reduction in the prevalence of the severe phenotype and a 96.9 ± 3.1 % decrease in lethality (Fig. 1c, e; Table 1).

### Post-treatment

The effectiveness of atropine and pralidoxime in protecting poisoned zebrafish was also tested using the post-treatment therapeutic approach (Fig. 1b). Although both drugs significantly reduced the prevalence of morphological changes, the effectiveness of pralidoxime was higher than that of atropine (Fig. 1f; Table 1). Furthermore, post-treatment with pralidoxime, but not atropine, significantly increased the survival of the larvae (Fig. 1d; Table 1).

Our next objective was to analyse the efficacy of the drugs targeting the secondary neuronal toxicity pathways in zebrafish. Thus, the efficacy of two NMDA receptor agonists (memantine and MK-801) and two dual-function NMDA receptor and AChR antagonists (caramiphen and benactyzine) was tested in zebrafish using the post-treatment therapeutic approach. Exposure of zebrafish larvae to selected concentrations of each drug alone did not affect mortality or morphology, with the exception of MK-801, which caused altered pigmentation in the larvae. Administration of the antiglutamatergic drugs significantly reduced both mortality and the prevalence of morphological changes in the head (Fig. 1d, f; Table 1). Post-treatment with MK-801 and caramiphen provided the maximal protection in this group, with a reduction of 63.9 and 60.1 % in mortality (Fig. 1d; Table 1), respectively, and a reduction in the prevalence of morphological changes of 81.4 and 72.0 % (Fig. 1f; Table 1), respectively.

Finally, the efficacy of two anti-inflammatory drugs, including one steroid (dexamethasone) and one non-steroid anti-inflammatory drug (ibuprofen), was tested in the severe acute OP intoxication zebrafish model using the post-treatment therapeutic approach. Although post-treatment of zebrafish exposed to 1 × LC_50_ CPO with both drugs increased the survival and decreased the prevalence of the severe phenotype, the degree of protection provided by ibuprofen was higher than that of dexamethasone (Fig. 1d, f; Table 1).

### Recovery of severe acute OP intoxication at different levels of organization

**Fig. 2:**
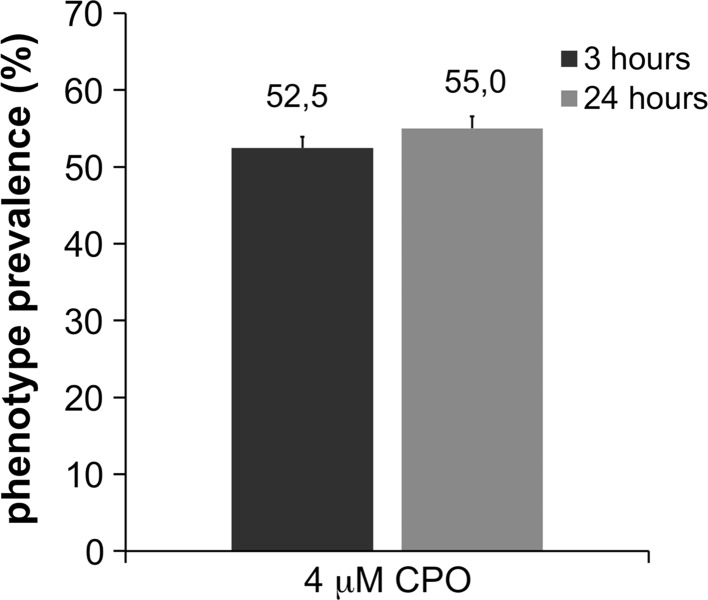
Prevalence of the changes in the head morphology observed 3 and 24 h post-exposure to 4 µM CPO. The results are representative of larvae from all recovery experiments. Responses are shown as % relative to the total number of larvae and represented as the mean ± SEM, total *n* > 300. *P* = 0.117, simple one-tailed Student’s *t* test

**Fig. 3:**
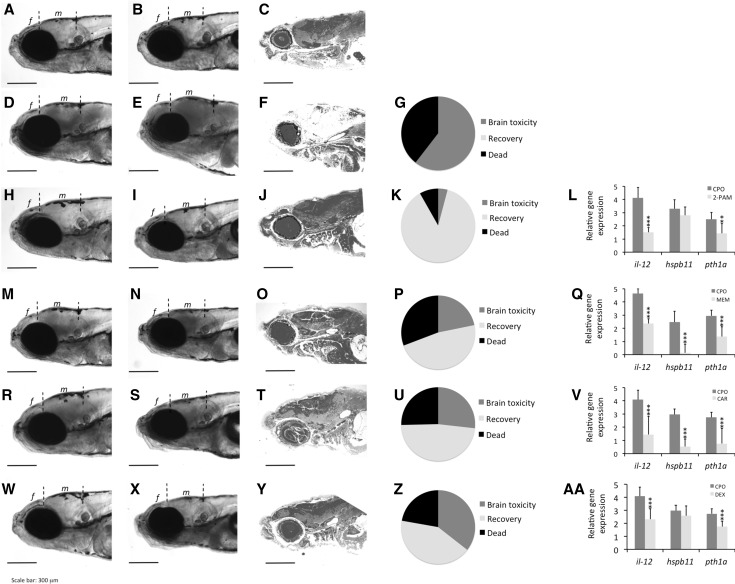
Recovery of the normal head phenotype in the severe acute OP intoxication zebrafish model after drug administration is predictive of the protective effect at the cellular and molecular levels. An adapted post-treatment protocol was used, and 7 days post-fertilization (dpf) larvae were exposed to 4 μM chlorpyrifos oxon (CPO) for 24 h. Then, the head morphology of each larva was analysed after 3 and 24 h of treatment. The *leftmost column of this panel* shows the lateral views of the head of one representative control larva (**a**) or a larva exposed to CPO for 3 h (**d**, **h**, **m**, **r**, **w**). Importantly, at 3 h post-exposure, only those larvae that exhibited signs of brain toxicity, including a mild enlargement of the forebrain (*f*) and midbrain (*m*), were selected for antidote administration. Thus, immediately after recording the 3 h post-exposure results, the antidotes were administered for an additional 21 h: 0.4 mM pralidoxime (I), 100 μM memantine (**n**), 25 μM caramiphen (**s**), and 40 nM dexamethasone (**x**). After analysis of the phenotypes at the end of the exposure, the larvae were fixed and processed for histopathological assessment. Parasagittal sections of the heads of larvae from the control (**c**), CPO (**f**), pralidoxime (**j**), memantine (**o**), caramiphen (**t**) and dexamethasone (**y**) groups at the end of the experimental period are shown. The control larva (**c**) has a normal histological structure of the central nervous system, but severe and extensive liquefactive acute damage was found after CPO exposure (**f**). Notice the absence of the extensive lesions induced by CPO exposure in larvae treated with the antidotes (**j**, **o**, **t**, **y**). In the three columns on the *left of this panel*, pictures from the same line correspond to the same animal. In addition, the efficacy of the antidotes can be assessed by the relative frequency of the three phenotypes: (1) severe phenotype (brain toxicity), (2) rescued phenotype and (3) dead. In the CPO (**g**) group, there was no recovery of the phenotype, but larvae treated with pralidoxime (**k**), memantine (**p**), caramiphen (**u**) and dexamethasone (**z**) exhibited a significant recovery. Finally, the rightmost column shows the effects of pralidoxime (**l**), memantine (**q**), caramiphen (**v**) and dexamethasone (**aa**) on the relative gene expression of *il*-*12*, *hspb11* and *pth1a*, three genes upregulated in zebrafish larvae exhibiting brain toxicity. *Asterisks* indicate significant differences between the larvae treated with a drug and those of the CPO group [*P < 0.05; **P < 0.01 or ***P < 0.001, following a one-tailed Student’s *t* test]. *Scale bars* 300 μm

Once the efficacy of the drugs was demonstrated in zebrafish, our next objective was to determine whether the protection against changes in head morphology was indeed predictive of a neuroprotective effect at the cellular and molecular levels. However, approximately 45 % of the larvae exposed to 1 × LC_50_ CPO for 24 h were resistant to the severe acute OP intoxication phenotype (Fig. 2). Thus, it is difficult to determine whether a larva with normal morphology following treatment with the drugs is a true “rescued” larva or just a “resistant” larva. To overcome this confounding factor, we used an adapted post-treatment approach protocol, in which the head morphology of each larva was analysed twice: (1) just before (i.e. 3 h after exposure to CPO) treatment and (2) at the end of the treatment. The protective effects of pralidoxime, memantine, caramiphen and dexamethasone in larvae exhibiting clear signs of severe acute OP intoxication at 3 h after exposure were also analysed at the end of the experiment (Fig. 3; Table 2).

**Table 2:** Relative frequency distribution of phenotypes after 3 and 24 h of exposure to 4 μM CPO

	Brain toxicity (%)	No brain phenotype (%)	Dead (%)	Total *n* ^a^
(a) Three hours post-exposure to 4 μM CPO (just before adding the antidotes)				
CPO	43.8 ± 2.9	44.8 ± 2.4	11.5 ± 3.0	336 (7)
Pralidoxime	38.0 ± 4.3	49.0 ± 4.5	13.0 ± 2.9	192 (4)
Memantine	40.6 ± 1.8	47.4 ± 3.0	12.0 ± 3.0	192 (4)
Caramiphen	37.0 ± 2.1	57.3 ± 1.3	5.7 ± 2.1	192 (4)
Dexamethasone	46.0 ± 3.0	47.9 ± 2.4	5.2 ± 0.6	192 (4)
(b) Twenty-four hours post-exposure to 4 μM CPO (21 h after adding antidotes): group of larvae exhibiting signs of brain toxicity at 3 h post-exposure				
CPO	57.3 ± 6.6	0.0 ± 0.0	42.7 ± 6.6	
Pralidoxime	3.8 ± 3.9	85.7 ± 7.1	10.4 ± 6.9	
Memantine	20.9 ± 8.9	48.2 ± 7.0	31.0 ± 5.9	
Caramiphen	25.7 ± 7.3	48.5 ± 8.3	25.8 ± 8.8	
Dexamethasone	35.0 ± 2.9	42.5 ± 1.4	22.5 ± 1.4	
(c) Twenty-four hours post-exposure to 4 μM CPO (21 h after adding antidotes): group of larvae with no signs of brain toxicity at 3 h post-exposure				
CPO	19.1 ± 2.8	66.7 ± 6.6	14.3 ± 4.5	
Pralidoxime	0.0 ± 0.0	92.4 ± 2.3	7.6 ± 2.3	
Memantine	8.4 ± 3.1	80.2 ± 5.5	11.4 ± 4.2	
Caramiphen	7.2 ± 2.8	73.7 ± 2.3	19.0 ± 4.4	
Dexamethasone	28.7 ± 4.3	62.1 ± 5.0	9.1 ± 6.5	

At 3 h post-exposure, immediately after determining the phenotype, four different antidotes were added, and the changes in the phenotype were recorded individually

^a^Number of experimental replicates

For each compound, Fig. 3 shows the phenotype of the head, at the gross morphological level, of one representative larva just before (3 h; Fig. 3a, d, h, m, r, w) and at the end (24 h; Fig. 3b, e, i, n, s, x) of the antidote treatment. At 3 h after exposure, larvae with moderate changes in head morphology (37–46 % of the surviving larvae; Table 2) were selected for the antidote treatment. When larvae were exposed to CPO for an additional 21 h, approximately 40 % died, and the remaining 60 % showed strong increases in the severity of the morphological changes in the head (Fig. 3e, g; Table 2). The histopathological evaluation of the brain of the same larva showed widespread liquefactive necrosis with a total disruption of the local architecture (Fig. 3f). Moreover, transcriptional analyses showed upregulation of genes related to calcium homeostasis (*hspb11*, *pth1a*) and the inflammatory response (*il*-*12*), molecular events potentially involved in severe acute OP intoxication pathogenesis (Fig. 3l, q, v, aa).

Pralidoxime had a very potent neuroprotective effect. Post-treatment with this drug recovered normal head morphology in 87.7 % of the selected larvae (Fig. 3h, i, k). Additionally, the lesions characteristic of severe acute OP intoxication at the histological level were not observed (Fig. 3j). Finally, pralidoxime also resulted in a partial recovery of *pth1a* and *il*-*12* levels (Fig. 3l).

Memantine also counteracted the effects of CPO on the gross morphology of the head in approximately 47 % of the larvae (Fig. 3m, n, p), and histopathological assessment showed the absence of general liquefactive necrosis (Fig. 3o). However, in this case, isolated or small groups of neurons displaying signs of acute degeneration and necrosis in different regions of the encephalon and also increased white areas in the white and grey matter indicated the presence of oedema (Fig. S1). Finally, memantine was shown to induce a significant recovery in the expression of all three genes (Fig. 3q). Interestingly, after treatment with memantine, the expression of *hspb11*, a calcium homeostasis-related gene, returned to the control levels (Fig. 3q). Very similar results were obtained with caramiphen, a dual-function AChR and NMDA receptor antagonist (Fig. 3r–v).

Finally, although dexamethasone protected against the liquefactive necrosis in approximately 42 % of the larvae (Fig. 3w–z), similar focal lesions as those observed following memantine treatment were present after treatment with this compound. Dexamethasone induced a significant recovery in only two of the three selected genes (Fig. 3a).

## Discussion

The present study demonstrates that a panel of drugs used to protect against the brain toxicity associated with severe acute OP intoxication in mammalian species exhibits similar neuroprotective effects in zebrafish. Therefore, these results indicate that the zebrafish model can be used to identify new compounds that protect against severe acute OP intoxication in humans.

The zebrafish model of severe acute OP intoxication used in the present study was developed using CPO, the major active metabolite of chlorpyrifos (CPF). While CPF is an atypical OP compound (López-Crespo et al. 2007), CPO is considered a prototypic OP compound (Faria et al. 2015; Garcia-Reyero et al. 2016). Thus, by selecting CPO for the model development, the specific effects of the parental compound as well as the effect of bioactivation were not considered. CPO is also a well-known developmental neurotoxicant (Estevan et al. 2013, 2014; Sogorb et al. 2016), and subacute exposure of zebrafish embryos to this compound inhibits the axonal growth of sensory neurons, primary motor neurons and secondary motor neurons (Jacobson et al. 2010; Yang et al. 2011). Although most structures of the nervous system of the zebrafish larvae are well developed at 7 days post-fertilization and the adverse effects found at different levels of organization are similar to those reported in adult mammals with severe acute OP intoxication, the possibility of adverse effects on the development of specific structures of the nervous system in the exposed larvae cannot be ruled out.

During severe acute OP intoxication, initial seizures rapidly progress to status epilepticus and finally result in profound brain damage (Shih and McDonough 1997; Tryphonas and Clement 1995). The proposed sequence of events consists of three phases: an early cholinergic phase, a transitional phase of mixed cholinergic/non-cholinergic modulation, and finally, a non-cholinergic phase (Shih and McDonough 1997). During the early cholinergic phase, AChE inhibition results in increased ACh levels in the synaptic clefts, triggering seizure activity in several susceptible areas of the brain. Then, seizure activity rapidly spreads, perturbing other neurotransmitter systems. Anticholinergic drugs, such as reversible AChE inhibitors and AChR antagonists, block seizures at this early stage and prevent brain damage. Consistent with the results in different mammalian models (Albuquerque et al. 2006; Grunwald et al. 1994; Lallement et al. 2002; Worek and Szinicz 1993), our results showed that pre-treatment of zebrafish with both peripherally (pyridostigmine) and centrally acting (physostigmine, huperzine A and galantamine) reversible AChE inhibitors provided significant protection against severe acute OP intoxication. The highest increase in survival was found after physostigmine pre-treatment, while the prevalence of morphological head changes was reduced to similar levels by all four tested drugs (Fig. 1c, e; Table 1). Interestingly, pyridostigmine, a peripherally acting compound, provided similar protection against brain toxicity as that of centrally acting compounds, indicating increased permeability of the brain–blood barrier (BBB) in our zebrafish model. Although the zebrafish and mammalian BBB both share structural and functional similarities (Fleming et al. 2013), zebrafish BBB maturation occurs between 3 and 10 dpf. The central neuroprotective effect of pyridostigmine could be related to the immaturity of the BBB in 7-dpf larvae, but a contribution of CPO to the increase in BBB permeability cannot be excluded (Parran et al. 2005).

Oximes reactivate the OP-inhibited AChE by dephosphorylating the enzyme active site. However, commonly employed pyridinium oxime reactivators, such as pralidoxime, HI-6 and obidoxime, are permanently charged and therefore show low penetration of the BBB. Consequently, for many years, it was assumed that the main therapeutic activity of oximes was via AChE reactivation in the peripheral nervous system (PNS) but not in the brain (de Koning et al. 2011). However, oximes may be active in the central nervous system (CNS) because several of these compounds prevent seizures and brain damage in nerve agent-exposed animals (Shrot et al. 2009). In the present study, we analysed the capacity of pralidoxime to counteract mortality and brain damage induced by acute exposure to 1 × LC_50_ CPO. Although it has been reported that pralidoxime can penetrate the BBB in a dose-dependent manner (Sakurada et al. 2003), the major effect reported in the literature is increased survival due to the reactivation of AChE in the PNS (Antonijevic and Stojiljkovic 2007; Quinby 1968). In our study, however, pre-treatment with pralidoxime decreased both the mortality rate and the prevalence of brain toxicity by approximately 97 %. This extremely potent neuroprotective action exhibited by pralidoxime in our zebrafish model suggests an increased permeability of the BBB. As discussed above, the immaturity of the BBB in 7-dpf zebrafish larvae, as well as the enhanced permeability induced by CPO, could be involved in the potent CNS activity of this oxime in our model. Although post-treatment with pralidoxime also provided significant protection against severe acute OP intoxication, the efficacy of pre-treatment was three- and sevenfold higher in increasing survival and decreasing brain toxicity, respectively (Fig. 1; Table 1). The higher efficacy of the pre-treatment compared to that of the post-treatment may be because the post-treatment effectiveness of the oximes is limited by the irreversible ageing of a portion of the inhibited AChE. Moreover, 3 h after exposure, seizures have already progressed to the transition phase, which is characterized by progressive activation of the glutamatergic system and decreased cholinergic control of the seizures. Our results suggest that new BBB-permeable oximes could be a part of the multifunctional drug therapy used to treat severe acute OP intoxication.

Secondary neuronal toxicity during severe acute OP intoxication results in brain damage (Kaur et al. 2014). We tested the zebrafish model using three groups of compounds that target key components of the pathophysiological pathways of secondary neuronal toxicity: glutamate antagonists, dual-function NMDA receptor and AChR antagonists, and anti-inflammatory drugs. The efficacy of MK-801, memantine, caramiphen and benactyzine has been demonstrated in a wide range of mammalian species and experimental protocols (Deshpande et al. 2010; McLean et al. 1992; Raveh et al. 2008; Raza et al. 2004; Zhou et al. 2005). We found that post-treatment of zebrafish with these drugs significantly decreased the prevalence of brain toxicity, with the highest protection provided by MK-801 and caramiphen. The use of anti-inflammatory drugs has been proposed to counteract neuroinflammation during severe acute OP intoxication (Amitai et al. 2006; Banks and Lein 2012; Dhote et al. 2007; Spradling et al. 2011). Post-treatment of the zebrafish with the anti-inflammatory agents ibuprofen and dexamethasone provided significant protection against brain toxicity, although this group of drugs was the least efficient of all tested treatments. Similar responses were observed in experiments conducted in rats (Amitai et al. 2006), suggesting that zebrafish could serve as a model to study inflammatory processes and their crucial roles in OP neurotoxicity.

The identification of chemical-specific gene expression signatures is very useful in determining the mode of action (MoA) of particular toxicants. Based on the transcriptional patterns observed in our previous work (Faria et al. 2015), we selected three genes involved in key pathophysiological pathways, including Ca^2+^ homeostasis (*hspb11* and *pth1a*) and the inflammatory pathway (*il*-*12*), of severe acute OP intoxication in zebrafish. The mRNA levels of the heat shock protein family B (small), member 11 (*hspb11*) were strongly upregulated following exposure to AChE inhibitors, an effect mediated by the increase in the intracellular calcium levels (Klüver et al. 2011). In the present study, the upregulation of *hspb11* found in the zebrafish larvae exposed to 1 × LC_50_ CPO was counteracted by post-treatment with memantine and caramiphen but not with pralidoxime and dexamethasone. The increase in intracellular Ca^2+^ appears to induce the *hspb11* upregulation and brain toxicity, and thus, these two drugs may provide neuroprotection to the zebrafish model of severe acute OP intoxication by blocking the Ca^2+^ entrance through the NMDA receptors.

Parathyroid hormone (PTH) is the major hormone that regulates calcium homeostasis. In turn, the synthesis and secretion of PTH is finely regulated by the serum calcium concentration, with hypocalcaemia resulting in a marked increase in the PTH transcripts (Moallem et al. 1998). The fact that drugs such as pralidoxime and dexamethasone, which are not directly linked to calcium homeostasis, partially counteract upregulation of *pth1a* in CPO-treated larvae suggests that serum calcium homeostasis can be restored by drugs that improve the general condition of the larvae.

A major hallmark of the inflammatory response is the release of cytokines and chemokines from activated macrophages. Acute intoxication with OP nerve agents can directly increase transcript and protein levels of pro-inflammatory cytokines (IL-12, IL-18) (Dhote et al. 2007; Johnson et al. 2011; Williams et al. 2003). Similar to previous studies in mammalian species, *il*-*12* expression significantly increased in untreated larvae. Interestingly, in spite of the different MoAs, all tested drugs decreased inflammation in our severe acute OP intoxication model. Because inflammation is a downstream event in the pathophysiological pathways of severe acute OP intoxication, these results suggest that drugs such as oximes and NMDA receptor antagonists, which target upstream events, can block the development of the inflammatory response.

Overall, the results from our study demonstrate that the zebrafish model of severe acute OP intoxication is highly predictive and can be used for identifying new compounds with therapeutic potential against the brain toxicity induced by severe acute OP intoxication in humans.

## Electronic supplementary material

Below is the link to the electronic supplementary material.

Supplementary material 1 (DOCX 6556 kb)
