# Supplementary material for: Zebrafish is a predictive model for identifying compounds that protect against brain toxicity in severe acute organophosphorus intoxication
Source: Arch Toxicol. 2016 Sep 21;91(4):1891–901. doi: 10.1007/s00204-016-1851-3 (PMC5364264; doi:10.1007/s00204-016-1851-3)
Supplement: Supplementary file 1 — Supplementary material 1 (DOCX 6556 kb) [file 204_2016_1851_MOESM1_ESM.docx]

**SUPPLEMENTARY INFORMATION**

**Zebrafish is a predictive model for identification of active compounds protecting against brain toxicity in severe acute organophosphorus intoxication**

Melissa Faria^1^, Eva Prats, Francesc Padrós, Amadeu M.V.M. Soares, Demetrio Raldúa

^1^To whom correspondence should be addressed. Email:

This file includes:

SI Methods ……………………………………………..2-4

SI Figures – Figure S1…………………………….…..5-5

SI Tables – Tables S1 and S2 ……………….………6-8

SI References ……………………..……..……..……. 9-10

**Supplementary Methods**

**Gross morphology analyses**

Larval head morphology was analysed with a Nikon SMZ1500 dissecting microscope at the end of the experiment. In experiments determining whether recovery in gross morphology of the head was predictive of a neuroprotective effect at lower levels of organization, morphology of the heads was also recorded after the initial 3 h of exposure to 1 x LC_50_ CPO prior to adding the antidotes. Images were acquired with a Nikon Digital Sight DSRi1 camera and NIS Elements AR software (version 3.0, NIKON Instruments INC, New York, USA) and saved as high-resolution (3849 x 3005 pixels) tagged image file format (TIFF). Control and treated larvae presenting clear recovery of head impairment and untreated larvae displaying a severe acute OP intoxication phenotype were collected for histopathological and transcriptional analyses.

**Histopathological evaluation**

Larvae were fixed in 10% phosphate-buffered formalin (pH 7.2) at room temperature. Subsequently, the larvae were dehydrated in a graded ethanol series (30, 50, 70, 90, 96 and 100%) for 30 min each. The larvae were progressively embedded in infiltration solution (Technovit® 7100, Kulzer) using absolute ethanol (1:3, 1:1 and 3:1) for 1 h in each step. Then, the larvae were placed in 100% infiltration solution overnight at room temperature. The specimens were placed in Teflon block moulding cups (Kulzer), and the remaining infiltration solution was removed using a pipette. New infiltration solution with hardener II (15:1) was added to the cups and placed in an incubator (37°C). After polymerization, hardened methacrylate blocks were glued to plastic holders using Technovit® 3040 (Kulzer) and removed from the cups. Blocks were sectioned with tungsten knives in a rotary microtome (Reichert-Jung). Sections (2 μm thick) were floated on a distilled water bath at room temperature, collected on clean glass slides and dried at 40°C. Then, the sections were immersed in an aqueous solution of 1% toluidine blue for 2 min and rinsed in tap water until the desired blue intensity and contrast with the background were obtained. The sections were dried at 37°C and mounted using DPX.

**RNA preparation and qRT-PCR analysis**

Total RNA was extracted from pools of 4 larvae using TRIzol Reagent (Invitrogen Life Technologies, Carlsbad, CA). RNA concentration was then measured by spectrophotometric absorption in a NanoDrop ND-8000 spectrophotometer (NanoDrop Technologies). After DNase I treatment (Ambion, Austin, TX), total RNA was reverse-transcribed to cDNA with a First Strand cDNA Synthesis Kit (Roche Diagnostics, Mannheim, Germany) according to manufacturer’s instructions. Real Time PCR was performed in a LightCycler ® 480 Real-Time PCR System (Roche Diagnostics, Mannheim, Germany) using SYBR Green PCR Master Mix (Roche Diagnostics, Mannheim, Germany). Cycling parameters were 95ºC for 15 min followed by 45 cycles of 95ºC for 10 s and 60ºC for 30 s. For each experimental condition, qPCR analyses of two independent experiments, with 4 or 5 biological replicates in each experiment and three technical replicates for each sample, were performed. Primers for the three selected genes (*il12a, hspb11, pth1a*) were designed using Primer Express 2.0 software (Applied Biosystems, Foster City, CA) and the Primer-Blast server (http://www.ncbi.nlm.nih.gov/tools/primer-blast; primer sequences in Table S2) and synthesized by Sigma. The housekeeping genes *ef1a* and *ppia2* were selected as reference genes (Oliveira et al. 2013).

Relative mRNA abundances of different genes were calculated from the second derivative maximum of their respective amplification curves (Cp, calculated by triplicates). To minimize errors in RNA quantification among different samples, Cp values for target genes (Cp_tg_) were normalized to the average Cp values for *ppia2*, used as reference gene, following Eq. (1)

ΔCptg = Cp*_ppia2_* − Cp_tg_

Changes in mRNA abundance in samples from different treatments were calculated by the ΔΔCp method (Pfaffl, 2001), using corrected Cp values from treated and non-treated samples (Eq. (2))

ΔΔCp_tg_ = ΔCp_tg__untreated − ΔCp_tg__treated

Fold-change ratios were derived from those values.

**Supplementary Figures**


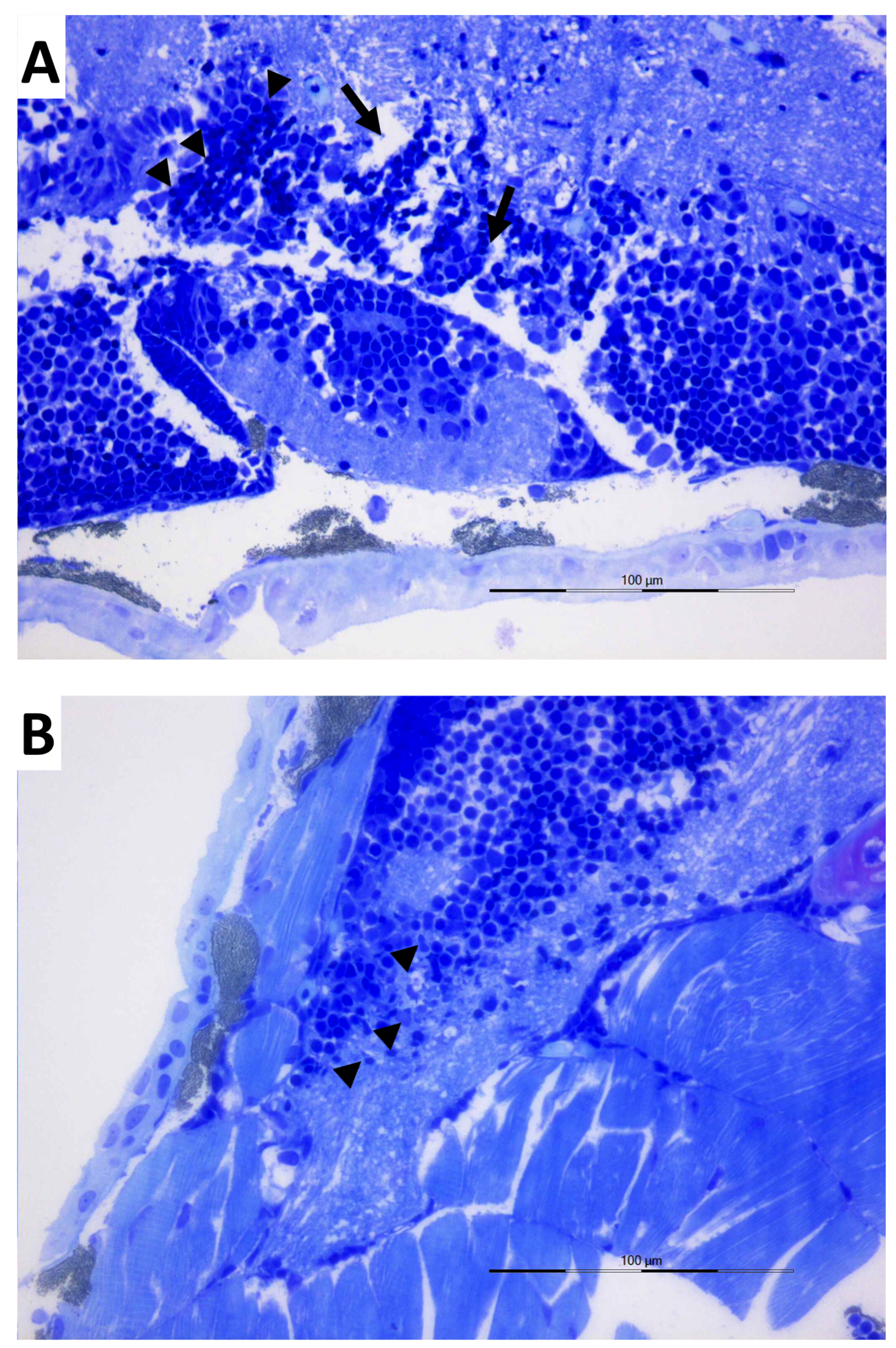


**Figure S1.** Parasagittal sections of the central nervous system of a larva exposed to 4 µM chlorpyrifos oxon (CPO) for 24 h and treated with memantine during the last 21 h of exposure (from 3-24 h of the CPO exposure). **A**. Focal necrosis (arrowheads) and slight edema (arrows) in the tissues. **B**. Focal necrosis (arrowheads) in the medulla oblongata.

**Supplementary Tables**

**Table S1.** Classification, CAS identifier, and medical use of the drugs tested in the zebrafish model of severe acute organophosphorus intoxication (*part 1*)

|  | **Drug class** | **CAS and purity %** | | **Medical uses** | **References** |
| --- | --- | --- | --- | --- | --- |
| Pralidoxime chloride |  | 51-15-0 | ≥97% |  |  |
|  | Standard antidotes for acute OP intoxication |  |  | Both are used as treatment for acute OP intoxication | Namba 1971 |
| Atropine |  | 51-55-8 | ≥99% |  |  |
|  |  |  |  |  |  |
|  |  |  |  |  | Čolović et al. 2013; Ma and Gang 2005; Yang et al. 2014 |
| Galantamine hydrobromide |  | 1953-04-4 | ≥98% | Alzheimer’s disease therapy |  |
|  |  |  |  |  |  |
| Physostigmine (eserine hemisulfate salt) | AChE reversible inhibitors | 64-47-1 | ≥99% | Alzheimer’s disease therapy |  |
|  |  |  |  |  |  |
| (±)-Huperzine A |  | 120786-18-7 | ≥98% | Alzheimer’s disease therapy |  |
|  |  |  |  |  |  |
| Pyridostigmine bromide |  | 101-26-8 | ≥98% | Muscle weakness, myasthenia gravis, prophylactic for acute OP intoxication - military | Jett and Yeung 2015 |
|  |  |  |  |  |  |
|  |  |  |  |  |  |
|  |  |  |  | Treatment of moderate-to-severe Alzheimer’s disease |  |
| Memantine hydrochloride |  | 41100-52-1 | ≥98% |  | Olney et al. 1989 |
|  | NMDA receptor antagonist |  |  |  |  |
| MK-801 |  | 77086-22-7 | ≥98% | Not clinically used | Foster et al. 1987 |
|  |  |  |  |  |  |

*Continue next page*

**Table S1.** Classification, CAS identifier, and medical use of the drugs tested in the zebrafish model of severe acute organophosphorus intoxication (*part 2*)

|  | **Drug class** | **CAS and purity %** | | **Medical uses** | **References** |
| --- | --- | --- | --- | --- | --- |
| Caramiphen hydrochloride |  | 125-85-9 | ≥98% | Anticonvulsant, antitussive, Parkinson's disease | Raveh et al. 2014 |
|  | NMDA and ACh receptor antagonist |  |  |  |  |
|  |  |  |  |  |  |
|  |  |  |  |  |  |
| Benactyzine hydrochloride |  | 57-37-4 | ≥98% | Antidepressant during 1960s, has been pulled off the US market | Shorter 2002 |
|  |  |  |  |  |  |
|  |  |  |  |  |  |
|  |  |  |  | Fever, treatment of mild-to-moderate pain | Both are included in the WHO Model List of Essential Medicines (19th WHO Model List of Essential Medicines (2015) |
| Ibuprofen |  | 15687-27-1 | ≥98% |  |  |
|  |  |  |  |  |  |
|  | Anti-inflammatory agents |  |  | Skin diseases, autoimmune conditions, allergies, asthma |  |
|  |  |  |  |  |  |
| Dexamethasone |  | 50-02-2 | ≥98% |  | [http://www.who.int/medicines/publications/essentialmedicines/EML2015_8-May-15.pdf).](http://www.who.int/medicines/publications/essentialmedicines/EML2015_8-May-15.pdf) |
|  |  |  |  |  |  |

**Table S2.** Sequences of primers used in this study

| Gene | ZFIN Acc number | GenBank Acc nº | Gene description | Sequence | | Amplicon length |
| --- | --- | --- | --- | --- | --- | --- |
|  |  |  |  |  |  |  |
| *ppia2* | ZDB-GENE-030131-8556 | BC062863.1 | 2-peptidylprolyl isomerase A | FW | 5'- GGGTGGTAATGGAGCTGAGA | 179 bp |
|  |  |  |  | RV | 5'- AATGGACTTGCCACCAGTTC | |
| *il12a* | ZDB-GENE-060724-1 | AB183001.1 | interleukin 12a | FW | 5'- GCTCTTCGTTTGACGACCGA | 80 bp |
|  |  |  |  | RV | 5'- GCGCTCTGTTGACGGTGAT | |
| *hspb11* | ZDB-GENE-030131-5148 | NM_001099427.1 | heat shock protein family B (small), member 11 | FW | 5'- CGCCTGCATCAGAGGATTTT | 81 bp |
|  |  |  |  | RV | 5'- TCCGAGCTGGAATGAAATGG | |
| *pth1a* | ZDB-GENE-040623-1 | NM_212950.1 | parathyroid hormone 1a | FW | 5'- GAGAAGCAAACGGGCAGTGA | 81 bp |
|  |  |  |  | RV | 5'- CAGTCCTGCCTCAGCTCCAC | |

**Supplementary References**

Čolović MB, Krstić DZ, Lazarević-Pašti TD, Bondžić AM, Vasić VM (2013) Acetylcholinesterase inhibitors: pharmacology and toxicology. Curr Neuropharmacol 11:315-335 doi: [10.2174/1570159X11311030006](http://dx.doi.org/10.2174/1570159x11311030006)

Foster AC, Gill R, Kemp JA, Woodruff GN (1987) Systemic administration of MK-801 prevents N-methyl-d-aspartate-induced neuronal degeneration in rat brain. Neurosci Lett 76:307-311. doi: 10.1016/0304-3940(87)90420-4

Jett DA, Yeung DT (2015) Strategies to enhance medical countermeasures after the use of chemical warfare agents on civilians. In: Gupta RC (ed) Handbook of toxicology of chemical warfare agents. Elsevier, Amsterdam, pp 1049-1056

Namba T, Nolte CT, Jackrel J, Grob D (1971) Poisoning due to organophosphate insecticides: Acute and chronic manifestations. Am J Med 50:475-492. doi: 10.1016/0002-9343(71)90337-8

Oliveira E, Casado M, Raldúa D, Soares A, Barata C, Piña B (2013) Retinoic acid receptors' expression and function during zebrafish early development. J Steroid Biochem Mol Biol 138:143-151 doi: [10.1016/j.jsbmb.2013.03.011](http://dx.doi.org/10.1016/j.jsbmb.2013.03.011)

Olney JW, Labruyere J, Price MT (1989) Pathological changes induced in cerebrocortical neurons by phencyclidine and related drugs. Science 244:1360-1362 doi: [10.1126/science.2660263](http://dx.doi.org/10.1126/science.2660263)

Pfaffl MW (2001) A new mathematical model for relative quantification in real-time RT-PCR. Nucleic Acids Res 29:e45-e45 doi: [10.1093/nar/29.9.e45](http://dx.doi.org/10.1093/nar/29.9.e45)

Raveh L, Eisenkraft A, Weissman BA (2014) Caramiphen edisylate: an optimal antidote against organophosphate poisoning. Toxicology 325:115-124. doi: [10.1016/j.tox.2014.09.005](http://dx.doi.org/10.1016/j.tox.2014.09.005)

Shorter E (2002) Looking backwards: a possible new path for drug discovery in psychopharmacology. Nat Rev Drug Discov 1:1003-1006. doi: [10.1038/nrd964](http://dx.doi.org/10.1038/nrd964)

Yang G, Wang Y, Tian J, Liu JP (2013) Huperzine A for Alzheimer's disease: A systematic review and Meta-analysis of randomized clinical trials. PLOS ONE 8:e74916 doi: [10.1371/journal.pone.0074916](http://dx.doi.org/10.1371/journal.pone.0074916)
